# An unusual case of hereditary transthyretin‐related amyloidosis and ulcerative colitis in a young Indian girl

**DOI:** 10.1002/jgh3.12206

**Published:** 2019-06-10

**Authors:** Vishal Sharma, Pankaj Sharma, Minu Singh, Roshan Agarwala, Kaushal K Prasad, Harshal S Mandavdhare, Usha Dutta, Prateek Bhatia

**Affiliations:** ^1^ Department of Gastroenterology Postgraduate Institute of Medical Education and Research Chandigarh India; ^2^ Department of Paediatrics Postgraduate Institute of Medical Education and Research Chandigarh India

**Keywords:** amyloidosis, hereditary, inflammatory bowel disease, transthyretin, ulcerative colitis

## Abstract

Hereditary transthyretin (TTR) amyloidosis is a multisystem disorder caused by extracellular amyloid deposition, usually presenting with neurological and cardiovascular involvement. Gastrointestinal involvement, if present, is usually in the form of motility symptoms like diarrhea, constipation, or diarrhea alternating with constipation. Presentations mimicking ulcerative colitis without other system involvement are rare. Here we present a case of a young female from northern India, who presented with blood‐admixed diarrhea without any feature of any other system involvement. She was diagnosed and treated as ulcerative colitis for two years with ambivalent response, although the compliance to therapy was also poor. She was re‐evaluated when she presented with recurrence of symptoms and new onset dysphagia. On evaluation, she was diagnosed as hereditary transthyrtetin related amyloidosis.

## Graphical abstract

Amyloidosis can occur as a consequence of inflammatory bowel disease or can mimic inflammatory bowel disease. Hereditary transthyretin (TTR) amyloidosis is hardly ever recognised in the Indian subcontinent. We report a case of TTR amyloidosis occurring in a young Indian girl who also had ulcerative colitis.

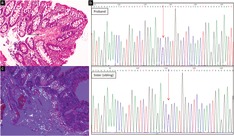

## Introduction

Hereditary transthyretin (TTR) amyloidosis is a multisystem disorder caused by extracellular amyloid deposition. It usually presents with neurological and cardiovascular involvement.^1^ Gastrointestinal involvement is commonly in the form of motility symptoms like diarrhea, constipation, or diarrhea alternating with constipation.^2^ Presentations mimicking ulcerative colitis without other system involvement are rare. In addition, there is geographical variation in the prevalence of TTR amyloidosis, with the most prevalent areas being Japan, Portugal, Sweden, and Brazil.^3^ TTR amyloidosis is rare in India, with only a few reported cases.^4^ Here, we report a case of a young girl from North India who presented with an isolated feature of inflammatory diarrhea without clinical evidence of cardiac or neurological involvement and was diagnosed with ulcerative colitis. After recurrence of symptoms associated with new‐onset dysphagia, an evaluation also helped recognize hereditary TTR amyloidosis.

## Case report

**Figure 1:**
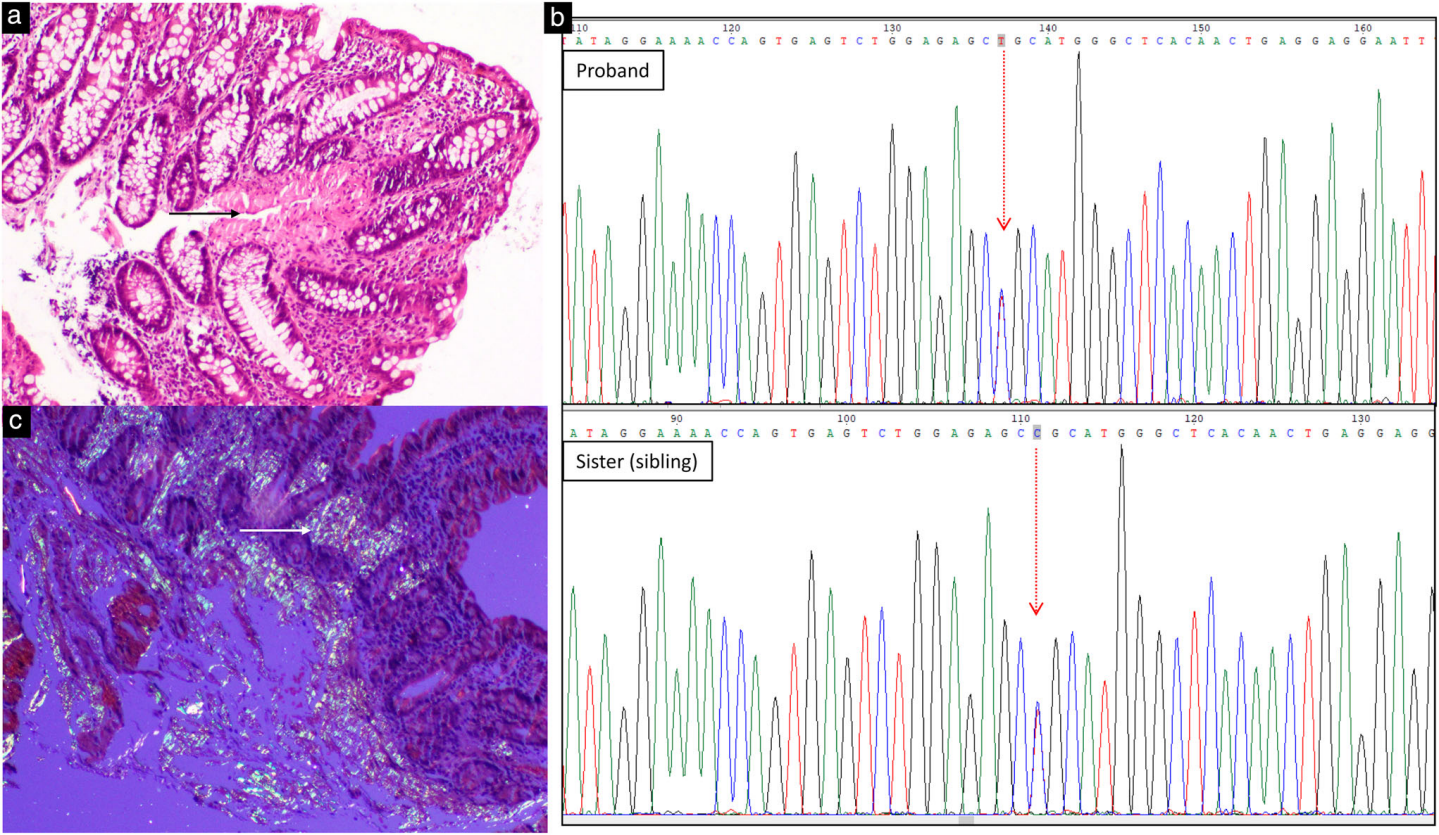
(a) Biopsy showing shortened and broadened villi with deposition of extracellular pinkish material in lamina propria and (b) apple‐green birefringence on Congo red staining and polarizing microscopy; (c) Sanger sequencing chromatogram showing heterozygous mutation with change in codon from T > C in exon 3 of the transthyretin gene in both the proband and the sibling at same position of codon.

A 20‐year‐old female was symptomatic with blood‐admixed loose stools off and on for 2 years. Her initial sigmoidoscopic evaluation in 2016 demonstrated loss of vascular pattern, granularity, friability, and superficial ulcerations in the rectosigmoid. A biopsy of rectosigmoid ulcers demonstrated features of chronic colitis with activity. Based on these findings, she was diagnosed with ulcerative colitis and started on mesalamine therapy. The response was ambivalent. She continued to take drugs but was lost to regular follow up. She presented again in 2018 with worsening symptoms associated with significant loss of weight and dysphagia. In the emergency department, she was managed for acute severe colitis with intravenous hydrocortisone and other supportive measures, to which she responded. Limited sigmoidoscopy was performed, which showed ulcerations and spontaneous bleeding from the rectum. Subsequently, she was further evaluated. Colonoscopy demonstrated loss of vascular pattern, hyperemia, and ulcerations involving majority of colon. In addition, an upper gastro‐intestinal endoscopy performed for dysphagia showed antral hyperemia and grooving in second part of duodenum. While rectal biopsy showed changes consistent with chronic colitis, the findings from the rest of colon, stomach, and duodenal regions showed deposition of extracellular eosinophilic material (Fig. 1a), which showed apple‐green birefringence on polarizing microscopy (Fig. 1b) on Congo red staining consistent with amyloidosis. However, we thought that the amyloid deposition could be either secondary to ulcerative colitis or primary amyloidosis. Further evaluation of the cause of amyloidosis was carried out. A review of clinical features demonstrated no history suggestive of neurological or cardiac involvement. However, her elder sibling (sister) had recently started having similar complaints and was under evaluation at a different tertiary care center but remained undiagnosed. Additional investigational work‐up of the index case demonstrated normal liver and renal function tests, normal contrast enhanced computed tomography abdomen, and normal serum and urine protein electrophoresis. However, echocardiographic evaluation showed left ventricular hypertrophy, and nerve conduction studies (right median, ulnar, peroneal, and tibial nerves) indicated features of sensorimotor axonal polyneuropathy in bilateral lower limbs. Considering the characteristic biopsy findings of apple‐green birefringence in blood vessels after Congo red staining, evidence of peripheral nerve, and cardiac involvement and the family history of sibling with similar complaints, a diagnosis of hereditary familial amyloidosis due to TTR protein deposition was suspected. The TTR gene was sequenced by Sanger sequencing and showed a pathogenic disease‐causing mutation in exon 3 (rs121918079; Chr/18: 31595143 T/C) with resultant amino acid change at position 55 from leucine to proline (Fig. 1c). Similar changes were also found in the gene analysis of her sister. However, gene analysis of her brother did not demonstrate the pathogenic mutation.

## Discussion

TTR amyloidosis is a systemic disorder characterized by the extracellular deposition of amyloid fibrils composed of TTR, a plasma transport protein for thyroxine and vitamin A, that is produced predominantly by the liver.^1^ About 120 different single or double mutations, or a deletion in the TTR gene, have been reported to date. Val30Met is the most common mutation.^1^ The mutation noted in the index case has been well characterized functionally and described in literature as related with early‐onset, aggressive, diffuse amyloidosis with cardiac and neurological involvement. McCutchen *et al*.^5^ have functionally shown that this mutation causes reduced stability of the TTR protein tetramer and increases its amyloidogenicity. It is important to recognize this condition since a targeted drug, Tafamidis, approved by the European Medicines Agency (EMA) is available for treatment and acts by stabilizing the tetramer structure of TTR.^6^ It has autosomal dominant inheritance with variable penetrance.^7^ In the index case, two of the three siblings tested showed the mutant allele.

Geographical variation in the prevalence of TTR amyloidosis has been well documented, with the most prevalent areas being Japan, Portugal, Sweden, and Brazil.^3^ TTR amyloidosis is rare in India, with only a few reported cases.^4^ Whether this is due to missed cases or reduced prevalence is a question that is unanswered.

Gastro‐intestinal tract (GIT) can be involved in primary amyloidosis, or it can result secondary to inflammatory bowel disease. However, the duration of primary disease in cases of secondary amyloidosis is usually long, which was not so in the index case. As such, primary amyloidosis was considered in the index case. Clinically, GIT involvement of the amyloid may present with a wide range of symptoms depending on the predominant site of involvement. Most commonly, it presents with motility symptoms (diarrhea, constipation, or diarrhea alternating with constipation), nausea, or vomiting.^2^ Isolated and predominant GI involvement is uncommon. The index case presented with predominant GI involvement with subclinical neurological and cardiac involvement. Hence, we should keep in mind the GI manifestations of amyloidosis, and any atypical presentation of ulcerative colitis should warrant consideration of alternate diagnoses. Family history is also key for the diagnosis of hereditary TTR amyloidosis. In addition, a meticulous examination of biopsies by an expert histopathologist is a must to observe the deposition of amyloid‐like substances. A confirmatory diagnosis in suspected cases should involve analysis for a genetic cause or immunocytochemical study of the amyloid‐positive tissue. Dysphagia in amyloidosis can be caused by multiple factors, including neuromuscular dysfunction.^8^ In hereditary TTR‐related amyloidosis, dysphagia is well recognized and believed to be related to combined involvement of the cranial nerves and enteric nervous system.^9^ Endoscopic changes in amyloidosis can be variable and may include hyperemia, erosions, granularity, nodularity, and plaque‐like deposits but can also be normal macroscopically.^8^ The endoscopic appearances are known to mimic inflammatory bowel disease. Interestingly, the rectal biopsy from the patient did not show evidence of amyloid deposition and only changes of chronic colitis, and therefore, a possibility of coexistence of ulcerative colitis with hereditary amyloidosis was entertained in this patient.
